# Initial stage of the biofilm formation on the NiTi and Ti6Al4V surface by the sulphur-oxidizing bacteria and sulphate-reducing bacteria

**DOI:** 10.1007/s10856-017-5988-2

**Published:** 2017-09-27

**Authors:** Beata Cwalina, Weronika Dec, Joanna K. Michalska, Marzena Jaworska-Kik, Sebastian Student

**Affiliations:** 1 Environmental Biotechnology Department, Silesian University of Technology, B. Krzywoustego 8, 44-100 Gliwice, Poland; 2 Institute of Industrial Organic Chemistry, Branch Pszczyna, Doświadczalna 27, 43-200 Pszczyna, Poland; 3 Faculty of Chemistry, Silesian University of Technology, B. Krzywoustego 6, 44-100 Gliwice, Poland; 4 Department of Biopharmacy, Medical University of Silesia, Jedności 8, 41-200 Sosnowiec, Poland; 5 Institute of Automatic Control, Silesian University of Technology, ul. Akademicka 16, 44-100 Gliwice, Poland

## Abstract

**Abstract:**

The susceptibility to the fouling of the NiTi and Ti6Al4V alloys due to the adhesion of microorganisms and the biofilm formation is very significant, especially in the context of an inflammatory state induced by implants contaminated by bacteria, and the implants corrosion stimulated by bacteria. The aim of this work was to examine the differences between the sulphur-oxidizing bacteria (SOB) and sulphate-reducing bacteria (SRB) strains in their affinity for NiTi and Ti6Al4V alloys. The biofilms formed on alloy surfaces by the cells of five bacterial strains (aerobic SOB *Acidithiobacillus thiooxidans* and *Acidithiobacillus ferrooxidans*, and anaerobic SRB *Desulfovibrio desulfuricans*—3 strains) were studied using scanning electron microscopy (SEM) and confocal laser scanning microscopy (CLSM). The protein concentrations in liquid media have also been analyzed. The results indicate that both alloys tested may be colonized by SOB and SRB strains. In the initial stage of the biofilm formation, the higher affinity of SRB to both the alloys has been documented. However, the SOB strains have indicated the higher (although differentiated) adaptability to changing environment as compared with SRB. Stimulation of the SRB growth on the alloys surface was observed during incubation in the liquid culture media supplemented with artificial saliva, especially of lower pH (imitated conditions under the inflammatory state, for example in the periodontitis course). The results point to the possible threat to the human health resulting from the contamination of the titanium implant alloys surface by the SOB (*A. thiooxidans* and *A. ferrooxidans*) and SRB (*D. desulfuricans*).

**Graphical abstract:**

## Introduction

Titanium and its alloys are useful for medical purposes, and also in many fields of industry. They are used for the production of implants and surgical instruments, as well as the pipelines for the oil transport, heat exchangers, aircraft and ships’ hulls, and many other elements and structures [1–3]. Ti-based alloys have higher strength properties in comparison to the pure titanium and to the other metal alloys, except the high-strength steels. They also have many other important properties that determine their broad applicability in technology and medicine. The special and remarkable properties of the titanium alloys may be obtained due to modification of the alloy surface [1–5]. NiTi and Ti6Al4V belong to important representatives of alloys usable for biomedical applications [4, 5].

The basic material used in the manufacture of an implant designed for a contact with a specific tissue (for example bone) should exhibit structural and mechanical properties similar to those of the tissue, while the surface of the material must be modified accordingly to ensure both the corrosion resistance and biocompatibility of the implant in the human body [1]. The improvement of safety and efficiency of the metal alloys usage in certain specific environments requires taking into account numerous factors of abiotic and biotic origin [1–3, 6, 7]. Amongst them, the susceptibility to the fouling of the implants made of Ti-containing alloys due to the adhesion of microorganisms and the biofilm formation is very significant [8–13], especially in the context of an inflammatory state induced by implants contaminated by bacteria [10]. The implants’ corrosion influenced by bacteria is also an important problem [10, 12, 13]—although titanium alloys are characterized by high corrosion resistance in natural and most industrial environments [1–3].

Bacteria of the *Acidithiobacillus* genus belong to the group of aerobic, acidophilic sulphur-oxidizing bacteria (SOB), which obtain energy for their growth and life activity from oxidation processes of the elemental sulphur and/or inorganic sulphur compounds [14]. *A. thiooxidans* and *A. ferrooxidans* species are considered to be especially corrosive in the aerobic environments as they can produce sulphuric acid and at the same time tolerate extremely high acidity of the environment [14, 15]. On the other hand, bacteria of the *Desulfovibrio* genus belong to the anaerobic sulphate-reducing bacteria (SRB). They obtain energy from the dissimilatory reduction of oxidized inorganic sulphur compounds (mainly sulphates), leading to the production of the hydrogen sulphide gas which is released into the environment [16]. These bacteria are often responsible for corrosion of various metals under anaerobic conditions [6, 7, 17]. Many SRB strains that showed particularly high corrosive aggressiveness in anaerobic environments belonged to the *D. desulfuricans* species [7, 17, 18]. Hence, both SOB and SRB are involved in the sulphur cycle in nature. They also play a role in many technological processes, in which they are purposely used (biotechnology), or as an unwanted factor partially responsible for the materials’ deterioration processes [16, 19, 20]. The literature data have shown that some of the bacterial species belonging to SOB and SRB may be responsible for the biofilm formation on the titanium alloys surface or even for their corrosion [15, 20, 21].

The aim of this work was to examine the differences between the bacteria strains belonging to the two above-mentioned groups in their affinity for the NiTi and Ti6Al4V alloys, and also—the extent to which the alloys tested can be vulnerable to colonization by the bacteria. For this purpose we analyzed progress in colonization of the alloy samples surface by the cells of five bacterial strains: one of each the species *A. thiooxidans* and *A. ferrooxidans*, and three strains of *D. desulfuricans*, using SEM and CLSM analyses. The protein concentrations in liquid media have also been analyzed. To our knowledge, this paper presents results of the comparative study of the SOB and SRB strains in terms of their abilities to the colonization of the Ti-containing alloys surface for the first time.

## Materials and methods

### Titanium-containing alloys

The NiTi alloy (Bimo Tech, Wrocław, Poland) used in this investigation was composed of 56.2 wt% Ni and 43.8 wt% Ti, and the Ti6Al4V alloy (Bimo Tech, Wrocław, Poland) contained 5.0 wt% Al, 4.3 wt% V, and 90.7 wt% Ti. The alloys in the form of rods were cut into cylinders with a diameter of 8 or 10 mm respectively, and a height of 4 mm. The surface of the samples was ground with abrasive silicon carbide papers (granulations 600 and 1000). Then, the samples were etched in a solution containing (g dm^−3^): HF 20, and H_2_SO_4_ 392, for 1 min, then rinsed with distilled water and cleaned ultrasonically in deionized water for 5 min. Afterwards the samples were electropolished (at 60 A dm^−2^ for 5 min) in a bath of the following composition (g dm^−3^): sulphuric acid 980, hydrofluoric acid 116, ethylene glycol 217 [22], and (additionally) acetanilide 102—in the case of the Ti6Al4V alloy. Next, the samples were rinsed using deionized water and subsequently treated with 99.8% ethanol for 1 h, and finally rinsed in sterilized distilled water.

### Organisms and culture

#### Sulphur-oxidizing bacteria

The WC1 strain of *A. thiooxidans* bacteria of high metabolic activity that is able to oxidize sulphur and its inorganic compounds [23] was cultured in the liquid culture medium of Waksman and Joffe containing (g dm^−3^): Na_2_S_2_O_3_∙5H_2_O 5.0, KH_2_PO_4_ 3.0, MgCl_2_∙6H_2_O 0.1, CaCl_2_∙6H_2_O 0.25, NH_4_Cl,0.1, FeSO_4_∙7H_2_O—traces; pH 4.0. The B1 strain of *A. ferrooxidans* bacteria that shows both the sulphur-oxidizing and iron-oxidizing activities [23] was cultured in the liquid culture medium 9K of Silverman and Lundgren composed of (g dm^−3^): (NH_4_)_2_SO_4_ 3.0, KCl 0.1, K_2_HPO_4_ 0.5, MgSO_4_∙7H_2_O 0.5, Ca(NO_3_)_2_ 0.01, FeSO_4_∙7H_2_O 44.2 (Fe^2+^ concentration: 9 g dm^−3^); pH 2.5 (by addition of 5 M H_2_SO_4_). All the chemicals used in experiments were of analytical reagent grade (Sigma-Aldrich). The SOB strains were cultured under aerobic conditions, in the Erlenmeyer flasks placed on a laboratory shaker, at 20–22 °C.

#### Sulphate-reducing bacteria

Three strains of the SRB that belong to *D. desulfuricans* species have been used in this study, namely: DSM642 standard strain isolated from soil (Swiss National Collection of Type Cultures), and two wild strains (DV/A and DV/B) deriving from patients suffering from various disorders of the gastrointestinal tract [24]. The SRB strains were cultured in Postgate’s culture medium containing (g dm^−3^): KH_2_PO_4_ 0.5, NH_4_Cl 1.0, CaCl_2_∙2H_2_O 0.01, MgCl_2_∙6H_2_O 1.0, FeCl_2_∙4H_2_O 0.003, sodium pyruvate 3.5, yeast extract 1.0 (pH 7.5), at 30 °C under anaerobic conditions (80% N_2_, 10% H_2_, and 10% CO_2_), using anaerobic chamber (MK 3 anaerobic workstation, dW Scientific, West Yorkshire, England).

### Media and test conditions

The culture medium appropriate for each SOB and SRB strain (Section 2.2) was used as the basic environment for the bacterial colonization tests. Additional studies were performed using the *D. desulfuricans* bacteria cultured in Postgate’s culture medium (5 cm^3^) supplemented with 10 cm^3^ of artificial saliva (I and II), because of the documented ability of this species to survive within the oral epithelial cells [25] and participation in the human periodontitis [26]. The artificial saliva I (modified Fusayama’s solution) was composed of (g dm^−3^): NaCl 0.7, KCl 1.2, Na_2_HPO_4_∙H_2_O 0.26, K_2_HPO_4_ 0.2, NaHCO_3_ 1.5, KSCN 0.33, and urea 0.13 (pH 6.7), whereas the artificial saliva II (modified Carter’s solution) contained (g dm^−3^): KCl 0.4, NaCl 0.4, CaCl_2_∙2H_2_O 0.795, Na_2_HPO_4_∙H_2_O 0.690, Na_2_S∙9H_2_O 0.005, and urea 1 (pH 3.7, adjusted with lactic acid) [27]. The low pH value of saliva II was meant to reflect the most unfavorable conditions occurring in the oral cavity after a meal when the pH value can be lower than 2.5 [28].

The alloy samples were immersed in an appropriate culture medium (alone or with the saliva addition in a volume ratio of 1:2; triplicates for every experimental set), which was inoculated with bacteria to obtain the concentration of the order of 10^6^ cells in 1 cm^3^. The SOB cells concentration in the culture medium was determined by Becton Dickinson (BD FACSAria III) flow cytofluorometer. The SRB cells concentration was monitored by optical density OD_436_ measurements. Studies concerning SOB were carried out under aerobic conditions whereas the SRB-containing samples were incubated under anaerobic conditions, at a temperature suitable for each bacteria strain (Sections 2.2.1 and 2.2.2). As control samples, the sterile systems without bacteria have been used. The bacterial growth and biofilm development were assessed after the different time of exposure to bacteria (from 5 min up to 48 h). The quantitative assessment of bacterial metabolic activity during the biofilm growth has been carried out by assay of protein concentration in the liquid medium and examination of bacterial biofilm by the use of SEM and CLSM microscopic analyses.

### Analysis of the protein concentration in liquid media

The bacteria cells affinity to the NiTi and Ti6Al4V alloys has been assessed quantitatively based on the determination of the protein concentration in the liquid culture at the early initial stage of biofilm formation on alloys samples (after 1 and 24 h). The protein concentration in liquid culture media was determined colorimetrically using BCA Protein Assay method (Thermo ScientificTM), developed for the colorimetric detection and quantification of the total protein amount. The aliquots of 0.05 cm^3^ of each liquid sample were taken and placed in Eppendorf safe-lock tube. Then the 1 cm^3^ of BCA Protein Assay working reagent was added to the tube, mixed well, and incubated at 37 °C for 30 min followed by cooling to room temperature. Finally, the absorbance at 562 nm was measured and the total protein concentration (mg cm^−3^) was determined using the standard curve.

### Microscopic examinations

#### Surface analysis using SEM

For biofilm imaging, the implant alloy samples after appropriate incubation with bacteria were rinsed carefully with a sterile phosphate buffered saline (PBS) solution to remove the dead and loosely attached bacteria. Then, the samples were fixed by standard fixation procedure in glutaraldehyde [29]. The prepared samples were all sputter-coated with gold and then analyzed with a scanning electron microscope. Some biodeterioration effects could be disclosed on the alloy surface under the biofilm layer after its removal using the ultrasonication. For this purpose, some samples were placed in a falcon tube with 50 cm^3^ of sterilized water, and then ultrasonicated (5 min at a maximum power of 30 W) using the Branson S-150 cell disruptor, which is useful for removing the corrosion products as well as biofilms. The surfaces after removal of the biofilms were finally rinsed with deionized water and dried in the air. The SEM studies were performed with the alloy samples exposed to appropriate liquid culture media inoculated with mentioned bacterial strains. For all the studies, Hitachi S-3400N SEM was used. The imaging areas were chosen to be representative of the entire surface of the samples.

#### Biofilm analysis using CLSM

Qualitative assessment of the living and dead bacteria present in the biofilm layer on alloy surface was performed by visualization of the biofilm constituents using the CLSM (CLSM Olympus Fluorview FV1000 Spectroscopic Confocal System), after 48 h incubation of both alloy samples with the bacteria *A. thiooxidans*. Samples covered with the bacteria *D. desulfuricans* were not investigated because of the inability to provide anaerobic conditions. The samples for CLSM studies were stained by BacLight® Live/Dead Viability Kit, which is a one-step assay for fluorescent staining of bacteria [30]. The test contains the nucleotide acid stains SYTO 9 and propidium iodide. SYTO 9 stains all bacteria, whereas the propidium iodide only penetrates the perforated membranes, and thus suppresses SYTO 9 fluorescence. Finally, bacteria with intact cell membranes were fluorescently stained green whereas bacteria with damaged membranes were fluorescently stained red. After the staining, samples were placed in a saline buffer solution and analyzed with a confocal laser scanning microscope. The imaging software Fluorview V 4.2 was used to process the CSLM images. The 3D images were first deconvoluted with Auto-quant × 3 (Media Cybernetics, Bethesda, MD) using an adaptive point spread function. Finally, 3D-reconstruction of the biofilm was created as volume rendered data sets using Imaris (Bitplane Scientific Software, Zurich, Switzerland).

## Results

### SOB and SRB growth on the alloys samples immersed in the culture media

**Fig. 1:**
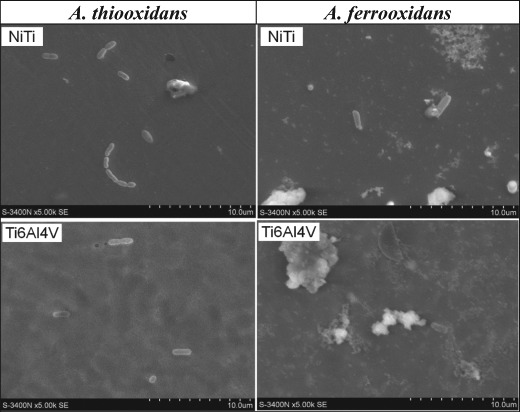
The SEM images of the NiTi and Ti6Al4V alloys surface with single cells of *A. thiooxidans* and *A. ferrooxidans* bacteria after 3 h incubation in the culture media

The SEM examinations concerning the *A. thiooxidans* and *A. ferrooxidans* bacteria presence on the NiTi and Ti6Al4V alloys surface after the 5 min, 15 min, 1 h, and 3 h incubation in the appropriate culture media shown only single bacterial cells adhered to the samples. For this reason, the SEM images obtained after 3 h have been presented in Fig. 1 as representational for this time as well as for shorter times of the incubation. The SOB cells (*A. thiooxidans* and *A. ferrooxidans*) were small—with a diameter of about 0.4–0.5 μm and lengths 1–2 μm, rod-shaped, straight with rounded ends. They were most often visible as single cells or occasionally in pairs, and sometimes in chains (Fig. 1, image *A. thiooxidans*—NiTi). Some cells are in a division stage (Fig. 1, image *A. thiooxidans*—Ti6Al4V). In this stage of biofilm formation by investigated SOB strains, only very weak and comparable bacteria affinities to both alloys tested were observed.

**Fig. 2:**
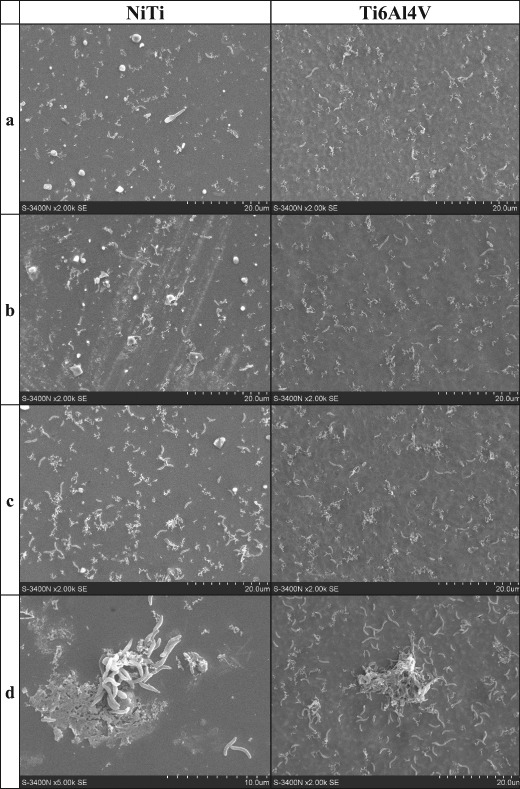
The SEM images of the NiTi and Ti6Al4V alloys surface colonized by *D. desulfuricans* bacteria after incubation in the culture media during: 5 min (**a**), 15 min (**b**), 1 h (**c**), and 3 h (**d**)

The SEM images showing colonization of the alloys surface by the *D. desulfuricans* strain are presented in Fig. 2. In this case, numerous bacterial cells were present on the surface of both alloys (Fig. 2a–d, NiTi and Ti6Al4V) and their abundance has increased with time. The SRB cells formed microcolonies visible after 1 and 3 h on the surface of both alloys tested. The cells of *D. desulfuricans* bacteria were of higher size as compared with SOB. We observed cells with a diameter of about 0.5–0.8 μm and lengths 1–3 μm (rarely much longer, even up to 10 μm). Cells were rod shaped with rounded ends, and they usually were curved (*vibrio*). Similarly to SOB, SRB were visible mainly as single cells, and sometimes in pairs or chains. At times, residues from the culture media were shown on the alloy surface (Figs. 1, 2).

**Fig. 3:**
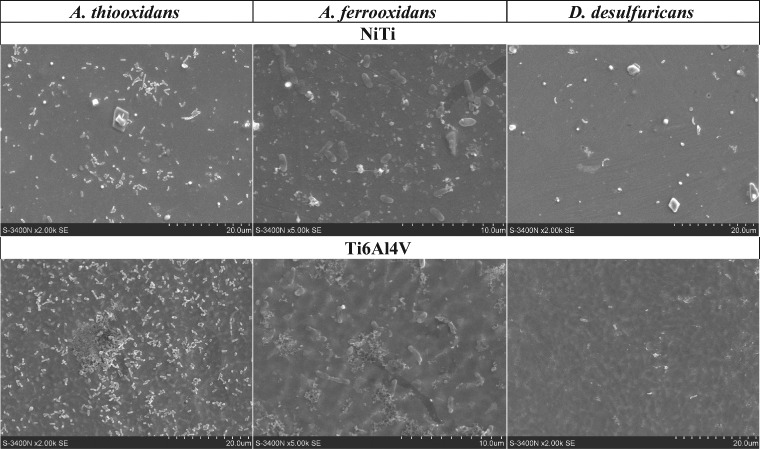
The SEM images of the NiTi and Ti6Al4V alloys colonized by bacteria *A. thiooxidans*, *A. ferrooxidans*, and *D. desulfuricans*, after 24 h incubation in the culture media

After 24 h of incubation (Fig. 3), the SOB cells (in particular of *A. thiooxidans* strain) were more numerous on the NiTi and Ti6Al4V alloys surfaces in comparison with the SRB *D. desulfuricans* strain. The last were visible in the Fig. 3 as singular cells, and no colonies—as opposed to numerous cells shown in Fig. 2.

**Fig. 4:**
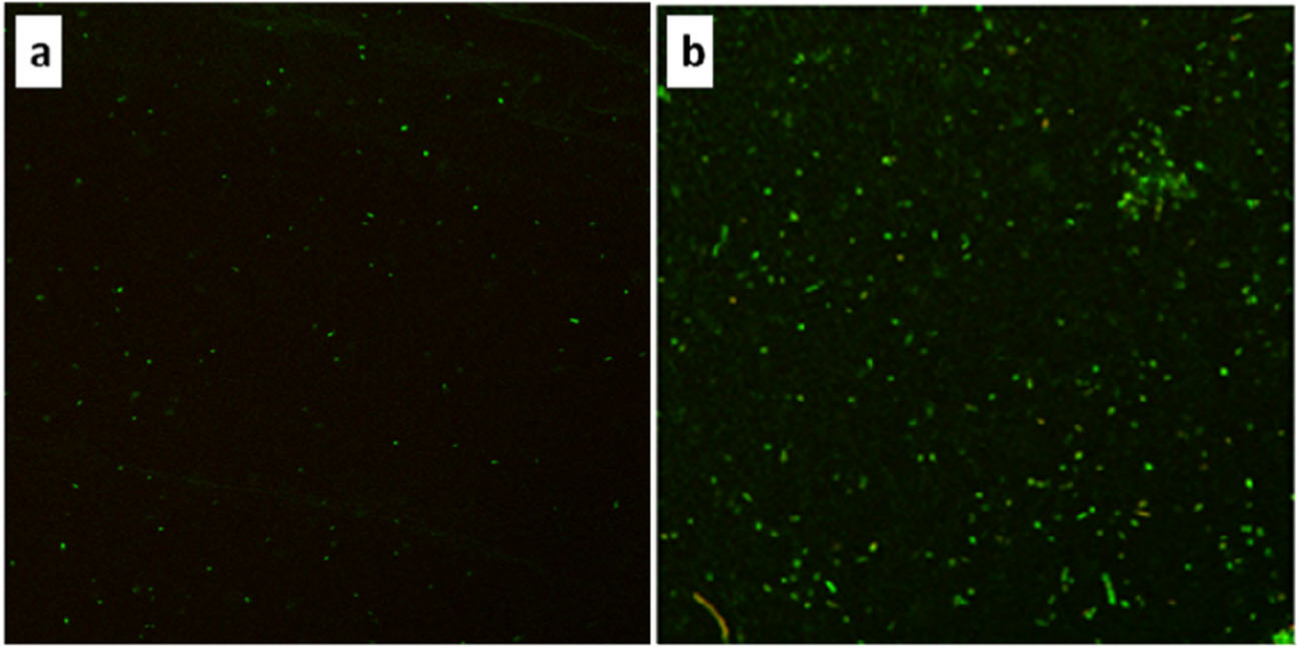
The CLSM analysis: images of fluorescently labeled cells of bacteria *A. thiooxidans* present on the NiTi (**a**) and Ti6Al4V (**b**) alloy surfaces, after 48 h incubation in the culture medium (optical zoom 600×, digital zooms: **a** 1.0×, **b** 1.7×) (color figure online)

**Fig. 5:**
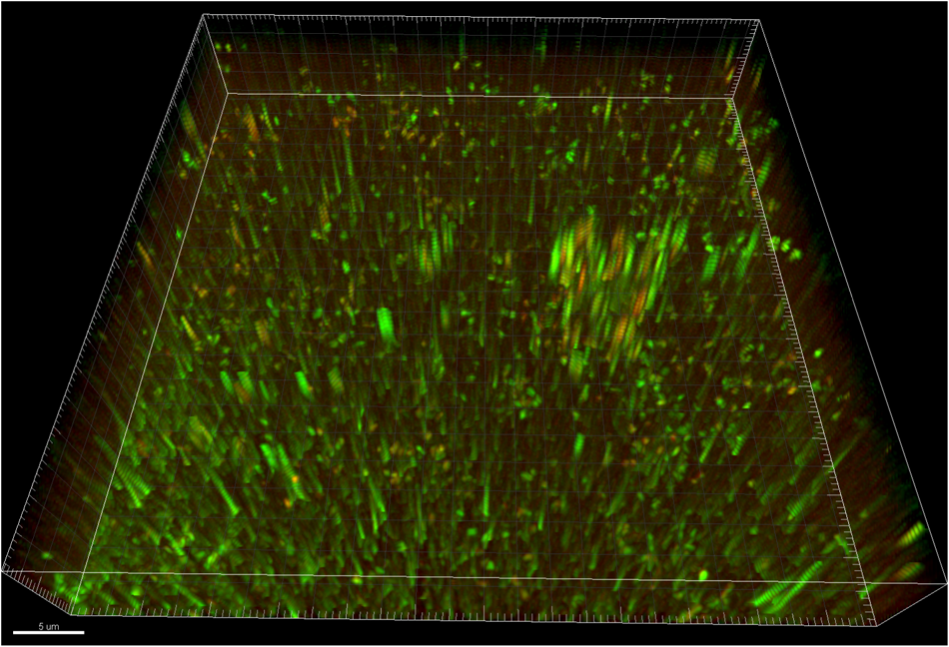
The 3D reconstruction of the 48-h biofilm (stained with BacLight® Live/Dead Viability Kit; living cells—green; dead cells—red) formed by *A. thiooxidans* bacteria on the Ti6Al4V alloy surface (based on scanning of optical cross-sections during the sample CLSM analysis, using the Imaris Bitplane Scientific Software) (color figure online)

The CLSM studies were carried out to verify the differentiation of the *A. thiooxidans* affinity to both alloys, as it was visible in Fig. 3, as well as to show the colony structure and explain how many bacterial cells that form biofilm are living or dead. Besides, the manner in which bacterial cells adhere to the surface was interesting due to the frequent observation of circular forms amongst bacterial cells. Results of the CLSM analyses have been presented in Figs. 4, 5. The numerous living cells of bacteria *A. thiooxidans* (fluorescently stained green) were present on surfaces of both NiTi (Fig. 5a) and Ti6Al4V (Fig. 5b) after the 48 h incubation. The cells were much more numerous on the Ti6Al4V alloy surface, and this corroborates the earlier observation (Fig. 3). A small number of dead cells (fluorescently stained red) were also present on both alloys. The 3D image of the biofilm structure formed by *A. thiooxidans* bacteria on the Ti-6Al-4V alloy surface after 48 h incubation is presented in Fig. 5. We can see numerous cells forming the biofilm of a few micrometers thickness, which are embedded in matrix of extracellular polymeric substances (EPS). The shape and size of vertical elements (Fig. 5) indicate that many cells were attached perpendicularly to the sample surface.

### SRB growth on the alloys samples in environment of the artificial saliva

**Fig. 6:**
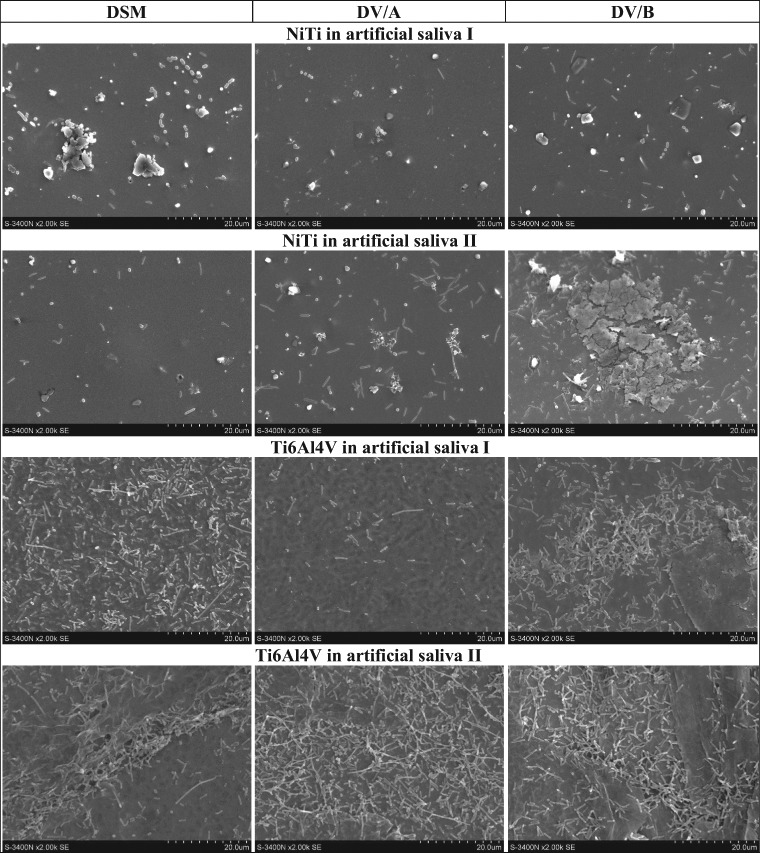
The SEM images of the NiTi and Ti6Al4V alloys surface colonized by bacteria *D. desulfuricans*, strains DSM, DV/A and DV/B, after 24 h incubation in the artificial saliva I and the artificial saliva II—imitating the conditions of inflammation

Taking into account the use of the titanium-containing alloys for dental and orthodontic purposes and possibility of their colonization by the SRB cells [10, 12, 26], studies have also been performed on three *D. desulfuricans* strains forming the biofilm on the surface of NiTi and Ti6Al4V implant alloys submerged in the solutions of artificial saliva (Section 2.3). The results obtained after 24 h incubation in these media (Fig. 6) showed considerably lower cells concentrations of all the tested strains on the surface of the NiTi alloy than on Ti6Al4V.

### The protein concentrations in the artificial saliva solutions

The protein concentrations in culture media supplemented with artificial saliva I and II during the short time bacteria incubation (from 1 to 24 h) with the NiTi are presented in Table 1. The results obtained after the first hour of the alloy incubation with SRB strains tested showed that concentrations of bacterial cells in research systems with artificial saliva I were lower (DSM ~36%, DV/A ~18%, DV/B ~14%) than in systems with artificial saliva II. During 24 h incubation, decrease in the cells concentration of all SRB strains tested was observed in both artificial saliva solutions. This effect was inconsiderable in case of the both artificial saliva systems with DSM strain, whereas it was more evident in research systems with DV/A and DV/B strains in artificial saliva I. It must be noted that the proteins concentrations in liquid media after 24 h represent effects resulting from the bacterial cells adhesion to solid surfaces as well as their growth and multiplication.

**Table 1:** The protein concentrations in the bacterial culture (PBC) of *D*. *desulfuricans* strains, and also in bacterial cultures supplemented with artificial saliva (PBCAS) I and II, after incubation for 1 or 24 h in the presence of the NiTi alloy

*D. desulfuricans* strain	PBC (μg cm^−3^)	Artificial saliva	PBCAS (μg cm^−3^)	
			Incubation time (h)	
			1	24
DSM	403.9 ± 7.6	I	283.6 ± 17.4	260.6 ± 5.6
		II	180.9 ± 21.4	170.9 ± 3.1
DV/A	402.6 ± 10.5	I	324.3 ± 50.2	190.9 ± 6.1
		II	266.6 ± 54.7	233.6 ± 28.8
DV/B	380.3 ± 8.7	I	343.9 ± 46.7	227.3 ± 21.2
		II	293.9 ± 9.5	236.3 ± 8.7

I—artificial saliva; II—artificial saliva imitating the inflammation conditions; PBC and PBCAS data are presented as arithmetic mean ± mean deviation

## Discussion

The SEM images of cells of the *A. thiooxidans*, *A. ferrooxidans* and *D. desulfurican* adhered to the NiTi and Ti6Al4V alloys surfaces (Figs. 1–3, 6) indicate that the observed morphological features of bacterial cells correspond to characteristics of species described in the Bergey’s Manual of Systematics of Archaea and Bacteria [31, 32]. However, sometimes we observed enormously elongated cells like those visible in the Fig. 6 in the case of the *D. desulfuricans* bacteria settled on the surface of Ti6Al4V alloy immersed in both artificial saliva tested (the artificial saliva I and the artificial saliva II—imitating the conditions of inflammation). The similar elongated bacterial cells were visible from time to time also in the SEM images showing the SOB biofilms. The elongated structures were more often observed when the exposure time was prolonged. It is known that bacteria can adopt several morphological types depending on the prevailing nutritional conditions as well as many other circumstances [33]. However, a general knowledge concerning these effects as well as the influencing factors remain insufficient to date.

Unexpected effects have been observed in the SEM images of the *A. thiooxidans*, *A. ferrooxidans* and *D. desulfurican* bacteria cells adhered to the NiTi and Ti6Al4V alloys surfaces, which were obtained after 24 h of incubation (Fig. 3). The numerous cells of both SOB species were present on the alloys surfaces in opposite to the SRB that were visible as singular cells. Observed effect may be the result of the detachment or even death of some *D. desulfuricans* cells due to unfavorable changes in the surrounding environment. In this case, the reason could also be the nutrient deficiency—in contrast to both autotrophic SOB strains that use CO_2_ as a carbon source. At the same time, an adaptation of the *A. thiooxidans* cells to altered environmental conditions probably occurred, thus allowing bacterial growth and their multiplication. Adaptability of the bacteria to the various adverse environments including but not limited to the high metal ions concentrations has been described for a variety of bacteria [34, 35]. Nevertheless, the adaptation effect may also concern *D. desulfuricans* bacteria as it can be seen in SEM images presented in Fig. 6. The dense biofilm of SRB has been formed after the 24 h incubation of the Ti6Al4V alloy in both the artificial saliva solutions. The relatively high SRB occurrence in the human oral cavity may also point to an adaptive response to altered environmental conditions [36].

The results concerning three *D. desulfuricans* strains forming the biofilms on the surface of NiTi and Ti6Al4V implant alloys when submerged in the solutions of artificial saliva, obtained after 24 h incubation in these media showed lower cells concentrations of all the tested strains on the surface of the NiTi alloy than on Ti6Al4V (Fig. 6). This may point to their lower affinity to the NiTi alloy samples immersed in the artificial saliva solutions or/and sensitivity to changes in the surroundings at this stage of growth under the conditions of the study. The observation seems to be confirmed by a decrease in the protein concentration in culture media supplemented with artificial saliva I and II during the short time bacteria incubation (1 h) with the NiTi (Table 1). The larger was the protein loss due to adhesion to the surface of the sample the higher was the bacteria cells affinity to the alloy surface (taking into account values of the arithmetic mean as well as the mean deviations, and assuming that the adhesion of bacterial cells to the vessel walls is constant for a given strain). On the other hand, the results of our previous studies [37] have shown the development of biofilm on the surface of NiTi during prolonged incubation (28 days). It can be supposed that the growth and multiplication of bacteria that form biofilms on solid surfaces under changing surroundings may have a sinusoidal course with the rising trend—due to the bacteria adaptation to the unfavorable conditions, but confirmation of this view needs further investigations. Some investigations point to the beneficial role of nickel ions on *D. desulfuricans* growth at Ni^2+^ concentrations up to 85.2 μM [38], or even 100 μM [39]. However, there exist conflicting data regarding the impact of the Ni^2+^ on bacterial adhesion to various biomaterials, including NiTi [40]. Besides, concentrations of Ni^2+^ leached from the NiTi implants to the surrounding environment have not been well known so far. This knowledge is very important as the presence of Ni^2+^ may cause risk of the inflammatory response in soft tissues [40], and *Desulfovibrio* bacteria can modulate inflammatory responses [25]. Thus, the next investigations are needed to elucidate these problems.

Results of the CLSM studies (Fig. 4) point to the *A. thiooxidans* bacteria vitality and corroborate the earlier observation made using SEM (Fig. 3), concerning the differentiation in affinity of this bacteria strain to both alloys. The cells were much more numerous on the Ti6Al4V alloy surface as compared to NiTi. Besides, the 3D image of Ti-6Al-4V alloy surface after 48 h incubation with *A. thiooxidans* bacteria (Fig. 5) indicate, that some cells have been attached perpendicularly to the sample surface, as it has been demonstrated by other authors [41–43]. It may be one of the reasons that sometimes the bacterial cells possessing longitudinal shape are visible in the microscopic (SEM) images as circular forms of the same diameter (for example—the *A. thiooxidans* cells in Fig. 1), suggesting falsely their spherical shape. This manner of the bacterial cells adhesion to various surfaces can be present due to specific properties of bacterial cells, the material surface as well as the surrounding environment [41–44].

The results presented in the study indicate that cells of both the aerobic SOB (*A. thiooxidans* and *A. ferrooxidans*) and anaerobic SRB (*D. desulfuricans*) may colonize the NiTi and Ti6Al4V alloys surface. Especially data concerning *D. desulfuricans* may point to the possible threat to the human health due to the contamination of the Ti-containing alloys surface by this bacteria species. It is recognized to be associated with human infections [26, 36, 45–47], and are able to survive within oral epithelial cells as well as to modulate the epithelial immune response, leading to initiation and progression of periodontal diseases [25]. Bacteria of the *Thiobacillus* genus that belong to the SOB group were only sometimes detected in the human oral cavity [21, 48, 49]. However, it already has been shown that many bacterial genera have been identified by DNA sequencing, but they were not detected using the microarray probes which did not target the 16S rRNA genes specific for the bacteria [49].

The biofilms formation on the NiTi and Ti6Al4V alloys surface by the strains of SOB and SRB may be of importance in the case of exposure to various external corrosive environments such as sea water or humid ground [15, 50]. A succession of microorganisms during the biofilm growth in any environment is commonly observed [6]. At the initial stage of the biofilm formation under natural conditions, it usually is composed of aerobic bacteria which consume oxygen present in the hydrogel layer. Their metabolic activity creates anaerobic conditions in a layer that remains in direct contact with the material. In this way, in the (micro)environment containing sulphur compounds, any material may be exposed either to the SOB and also SRB. Even if some autotrophic microorganisms do not cause corrosion, they may influence the biofilm formation due to transformation the inorganic carbon (CO_2_) into the organic forms useful for the other microorganisms that may cause corrosion.

## Conclusions

The results of the SEM and CLSM studies and the biochemical analysis indicate that the NiTi and Ti6Al4V alloys may be colonized by aerobic SOB of the species *A. thiooxidans* and *A. ferrooxidans*, as well as by anaerobic SRB of *D. desulfuricans* species. Thus both groups of bacteria of the sulphur cycle may be responsible for the deterioration processes of the implant alloys tested. In the initial stage of biofilm formation, the higher affinity of SRB for both alloys has been documented. However, the SOB strains have indicated the higher (although differentiated) adaptability to changing the environment as compared with SRB. Stimulation of the SRB growth on the NiTi and Ti6Al4V alloys surface was observed during incubation in the liquid culture media supplemented with artificial saliva, especially of lower pH (imitated conditions under the inflammatory state, for example in periodontitis). The results point to the possible threat to the human health resulting from the contamination of both implant alloys surface by the SOB (*A. thiooxidans* and *A. ferrooxidans*) and SRB (*D. desulfuricans*).
